# Adverse event profile differences between pralsetinib and selpercatinib: a real-world study based on the FDA adverse events reporting system

**DOI:** 10.3389/fphar.2024.1424980

**Published:** 2024-09-20

**Authors:** Qiong Jie, Yuanyuan Li, Li Jing, Jinjin Chen, Yang Li

**Affiliations:** Department of Pharmacy, Nanjing First Hospital, Nanjing Medical University, Nanjing, China

**Keywords:** pralsetinib, selpercatinib, adverse drug reactions, FAERS, pharmacovigilance

## Abstract

**Aims:**

The objective of this study is to compare the adverse events (AEs) associated with pralsetinib and selpercatinib.

**Methods:**

To evaluate the imbalance of AEs linked to pralsetinib and selpercatinib in real-world data, the reporting odds ratio (ROR) was utilized to detect potential signals of AEs. Stratified analysis was conducted to examine the differences in AEs occurring among different genders and age groups taking pralsetinib and selpercatinib.

**Results:**

FAERS received 891 reports for pralsetinib and 569 reports for selpercatinib. Our analysis confirmed expected AEs like hypertension, fatigue, and elevated transaminase levels. Unexpected AEs such as rhabdomyolysis, myocardial injury and cognitive disorder were associated with pralsetinib, while selpercatinib was linked with pulmonary embolism, deep vein thrombosis, and pericardial effusion. The risk of AEs such as decreased platelet count, anemia, decreased white blood cell count, pneumonitis, asthenia, and edema caused by pralsetinib is significantly higher than that of selpercatinib. In contrast, the risk of AEs such as ascites, elevated alanine aminotransferase, and elevated aspartate aminotransferase caused by selpercatinib is significantly higher than that of pralsetinib. Women treated with pralsetinib experience higher rates of hypertension, pulmonary embolism, and blurred vision than men, who are more susceptible to rhabdomyolysis. Adults between 18 and 65 years are more likely to experience taste disorder, edema, and pulmonary embolism than individuals older than 65, who are particularly vulnerable to hypertension. For patients treated with selpercatinib, males demonstrate a significantly higher incidence of QT prolongation, urinary tract infection, and dysphagia. Individuals aged 18 to 65 are more likely to experience pyrexia and pleural effusion than those older than 65, who are more prone to hypersensitivity.

**Conclusion:**

In the clinical administration of pralsetinib and selpercatinib, it is crucial to monitor the effects of gender and age on AEs and to be vigilant for unlisted AEs.

## 1 Introduction

The rearranged during transfection (RET) proto-oncogene is thoroughly studied and is essential for multiple physiological and developmental functions. RET rearrangements are present in about 10%–20% of thyroid cancers and 1%–3% of non-small cell lung cancer (NSCLC) cases, making routine testing for RET fusions at diagnosis advisable (Nacchio et al., 2022; Li et al., 2019). Traditional multitarget kinase inhibitors have treated RET fusion-positive lung cancers but show limited efficacy (Gautschi et al., 2017; Takeuchi et al., 2021). In the past, the treatment plans for advanced lung cancer with RET fusion were similar to those for NSCLC without oncogenes. Lately, the development of selective RET tyrosine kinase inhibitors has been changing, creating a new model in personalized healthcare.

In 2020, pralsetinib and selpercatinib, both RET inhibitors, received approval from the FDA in the United States. Data from the worldwide, multi-site Phase I/II ARROW study showed that pralsetinib had a strong overall response rate and extended median progression-free survival in patients who had received treatment before (61%; 17.1 months) and those who had not (70%; 9.1 months) (Griesinger et al., 2022). Similarly, an analysis of the phase I/II LIBRETTO-001 trial showed that selpercatinib had an objective response rate of 64% in previously treated NSCLC patients with RET fusion and 85% in patients who had not received treatment before (Subbiah et al., 2022). Given the widespread clinical use of pralsetinib and selpercatinib, comprehending the incidence and severity of their AEs is essential. However, current clinical evidence might not fully capture the real-world safety profile of pralsetinib and selpercatinib.

FAERS, the FDA Adverse Event Reporting System, is a well-known system for reporting drug-related adverse events and medication errors (Fang et al., 2014). The FAERS database is utilized to evaluate possible associations between drugs and AEs during post-marketing surveillance and plays a significant role in pharmacovigilance research. Our objective in this research was to examine the safety characteristics of the RET inhibitors pralsetinib and selpercatinib by analyzing data from the FAERS database. Concurrently, we will perform a stratified analysis of selected AEs by gender and age to assess the variation in adverse event frequencies among populations treated with pralsetinib or selpercatinib.

## 2 Materials and methods

### 2.1 Date source

Our study focused on the RET inhibitors pralsetinib and selpercatinib. Reports from the FDA’s FAERS database, spanning 2020Q2 to 2023Q3, were acquired for analysis. Due to non-standardized drug naming in the FAERS database, we used both generic (pralsetinib, selpercatinib) and brand names (Gavreto, Retevmo) as search keywords. Our analysis was limited to reports identifying pralsetinib and selpercatinib as the primary suspect drugs. Additionally, we employed MedDRA version 26.1 to automate AE classification into System Organ Class (SOC) and Preferred Term (PT) for heightened specificity. SOC and PT are widely recognized and utilized for analyzing FAERS data.

### 2.2 Data cleaning

This study analyzed reports containing three key elements: identifiable patients, suspected drugs, and AEs. Following FDA guidelines, we removed duplicate reports and used the most recent case numbers for disproportionality analysis. AEs not related to the drugs, such as medication errors, therapeutic procedures, administration errors, and disease progression, were excluded. Therefore, our analysis focused exclusively on drug-induced AEs, disregarding those linked to the disease state itself.

### 2.3 Data mining and statistical analysis

Disproportionality analysis is an established method that compares the frequency of AEs for a specific drug against all other drugs. Additionally, this method can detect statistical signals of associations between drugs and AEs. The RORs were calculated to uncover signals suggestive of an increased risk of drug-related AEs with pralsetinib and selpercatinib. A higher ROR signifies a greater disproportionality and a stronger signal, suggesting that the drug in question may have a higher likelihood of causing a particular AE compared to other drugs. Furthermore, this study analyzed the impact of different genders and ages on AEs caused by pralsetinib and selpercatinib treatments. Chi-square tests was used to analyze the clinical features of AE reports and compare them between the two medications, *p* < 0.05 indicates that the difference is statistically significant.

## 3 Results

### 3.1 Descriptive analysis

From 1 May 2020, to 30 September 2023, the FAERS database recorded a total of 5,415,314 reports, following the removal of duplicates. Out of these reports, 891 identified pralsetinib as the primary suspect drug, associating it with 3,064 AEs. For selpercatinib, there were 569 reports with 1,237 AEs associated. The clinical characteristics of pralsetinib and selpercatinib are compared in Table 1. Differences were observed in patient age, reporter type, and reporting country between the pralsetinib and selpercatinib groups.

**TABLE 1:** Comparison of clinical characteristics between reports with pralsetinib and selpercatinib

	Pralsetinib (n,%)	Selpercatinib (n,%)	*p*-value
Number of events	891	569	
Gender			0.448
Female	452 (50.73%)	271 (47.63%)	
Male	343 (38.50%)	228 (40.07%)	
Missing	96 (10.77%)	70 (12.30%)	
Age (year)			0.001
<18	2 (0.22%)	2 (0.35%)	
18–65	260 (29.18%)	126 (22.14%)	
≥65	262 (29.41%)	150 (26.36%)	
Missing	367 (41.19%)	291 (51.14%)	
Reporter type			<0.001
Consumer	594 (66.67%)	258 (45.34%)	
Physician	177 (19.87%)	146 (25.66%)	
Pharmacist	30 (3.37%)	23 (4.04%)	
Health professional	86 (9.65%)	98 (17.22%)	
Unknown	4 (0.45%)	44 (7.73%)	
Reporter country			<0.001
United States	482 (54.10%)	374 (65.73%)	
Japan/Canada	283 (31.76%)	67 (11.78%)	
Other countries	126 (14.14%)	128 (22.49%)	

In the reports concerning pralsetinib, females comprised a greater proportion (50.73%) compared to males (38.50%). The majority of patients reported were over the age of 65, with a median age of 71. More than half of the reports (54.10%) came from the United States and were primarily provided by customers (66.67%). Lung cancer (59767.0%), thyroid cancer (141, 15.8%), and other solid malignancies (293.3%) were the most commonly reported indications, which did not fully align with FDA-approved uses. Table 2 illustrates that hospitalizations represented the largest share of serious outcomes at 29.18%, followed by 254 cases designated as other serious outcomes (28.51%) and 130 death events (14.59%).

**TABLE 2:** Comparison of serious outcomes between reports with pralsetinib and selpercatinib from the FAERS database (2020Q2 to 2023Q3).

Serious outcomes	Pralsetinib n (%)	Selpercatinib n (%)	*p*-value
Hospitalization	260 (29.18%)	128 (22.50%)	0.005
Disability	7 (0.79%)	5 (0.88%)	1.000
Life-threatening	13 (1.46%)	13 (2.28%)	0.245
Death	130 (14.59%)	83 (14.59%)	0.999

The selpercatinib data indicated disparities when compared to pralsetinib, including age composition, reporter type, and reporting country. Gender distribution, however, was similar, with males accounting for 40.07% of the reports. Lung cancer (283 cases, 49.6%), thyroid cancer (100 cases, 17.6%), and other solid tumors (11 cases, 1.9%) emerged as the top three reported indications, matching FDA-approved uses. Hospitalization rates for severe AEs were notably higher for pralsetinib compared to selpercatinib, as demonstrated in Table 2 (29.18% vs. 22.50%, *p* = 0.005).

### 3.2 Disproportionality analysis

**FIGURE 1:**
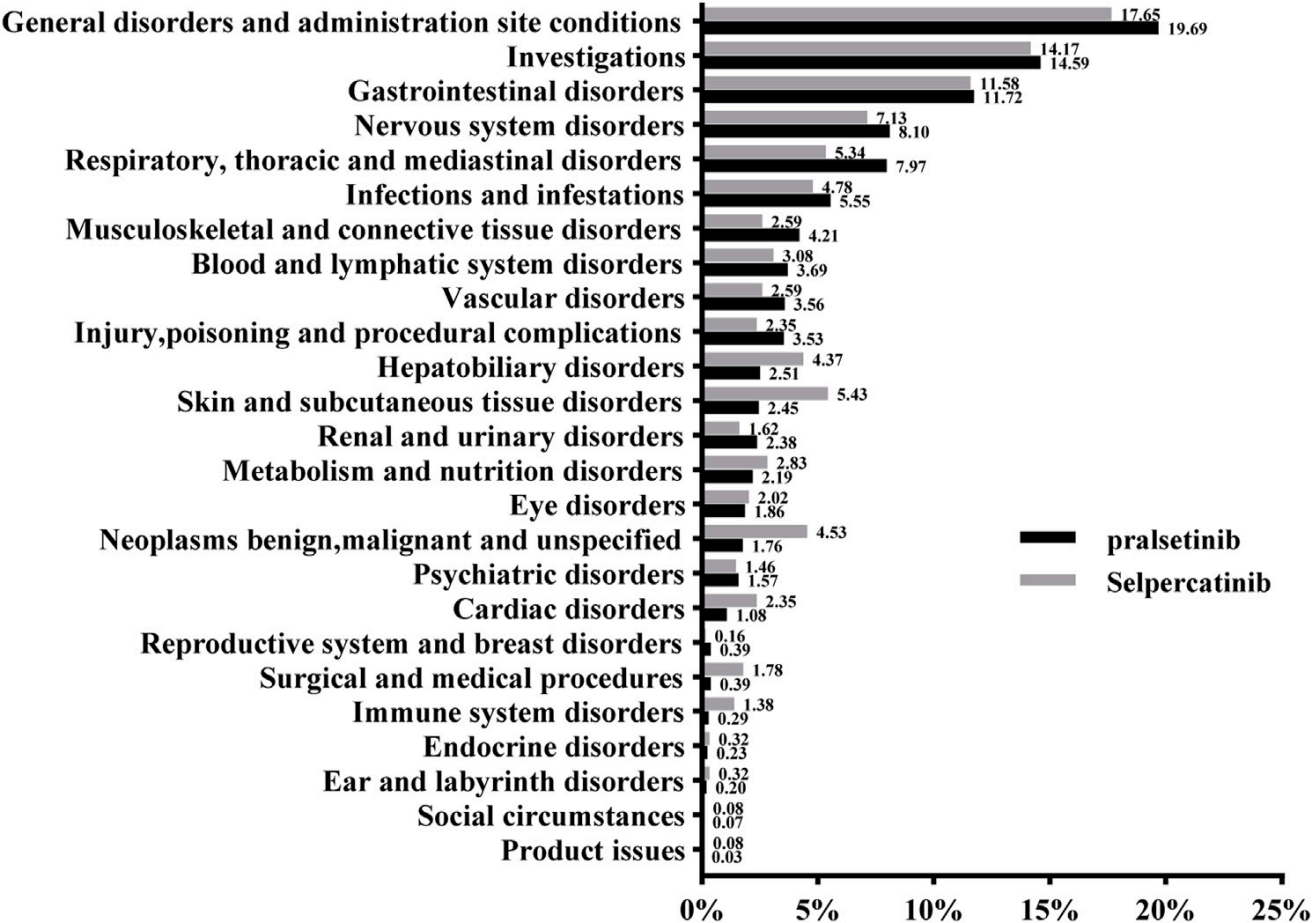
The proportion of adverse events related to pralsetinib and selpercatinib at the SOC level.

Figure 1 included information on the reports of pralsetinib and selpecatinib at the SOC level. Statistical analysis revealed that pralsetinib-induced AEs occurred in 25 SOCs. Within these, several SOCs were identified as significant, meeting the ROR criteria of three or more cases. Significant SOCs included general disorders (603 cases, 19.68%), investigations (447 cases, 14.59%), gastrointestinal disorders (359 cases, 11.72%), nervous system disorders (248 cases, 8.10%), respiratory, thoracic, and mediastinal disorders (244 cases, 7.97%), blood and lymphatic system disorders (113 cases, 3.69%), vascular disorders (109 cases, 3.56%), hepatobiliary disorders (77 cases, 2.51%), and renal and urinary disorders (73 cases, 2.38%) as shown in Supplementary Table 1. Similarly, selpercatinib-induced AEs were also concentrated in 25 SOCs Supplementary Table 2. Significant SOCs for selpercatinib that met the algorithm criteria included investigations (175 cases, 14.17%), gastrointestinal disorders (143 cases, 11.58%), hepatobiliary disorders (54 cases, 4.37%), blood and lymphatic system disorders (38 cases, 3.08%) and metabolism and nutrition disorders (35 cases, 2.83%) Supplementary Table 2. Significant differences between both drugs were observed in SOCs such as investigations, gastrointestinal disorders and blood and lymphatic system disorders (Table 3).

**TABLE 3:** RORs and *p*-value for pralsetinib and selpercatinib at SOC level.

SOC of interest	Pralsetinib		Selpercatinib		*p*-value
	N = 891	ROR (95%CI)	N = 569	ROR (95%CI)	
Investigations	447	2.71 (2.45–2.99)	175	2.62 (2.23–3.07)	<0.001
Gastrointestinal disorders	359	1.58 (1.42–1.77)	143	1.56 (1.31–1.85)	<0.001
Blood and lymphatic system disorders	113	2.22 (1.84–2.67)	38	1.84 (1.33–2.54)	<0.001
Hepatobiliary disorders	77	3.23 (2.58–4.05)	54	5.68 (4.33–7.47)	0.580

Following the implementation of the ROR algorithm, our study identified 103 indications of AEs caused by pralsetinib within 17 SOCs. Most of the AEs mentioned were already included in the pralsetinib label, including hypertension, constipation, diarrhea, fatigue, oedema, fever, cough, and dry mouth. Furthermore, AEs related to blood cells, such as lymphopenia, reduced neutrophils, anemia, and decreased platelet count, were also observed. Our data analysis revealed some unexpected AEs, such as myocardial necrosis marker increased (ROR = 246.41), cognitive disorder (ROR = 4.30), balance disorder (ROR = 3.78), hypogeusia (ROR = 146.79), rhabdomyolysis (ROR = 2.73), dysphonia (ROR = 4.87), cerebral infarction (ROR = 4.68), vision blurred (ROR = 2.73), and myocardial injury (ROR = 27.75). In general, our results indicate that pralsetinib could result in various AEs, some of which are not currently listed on the label.

Upon comparing the notable AEs listed or not listed in the labels of pralsetinib and selpercatinib, it was determined that pralsetinib had a notably greater amount of reported AEs linked to hypertension, fatigue, low red blood cell count, decreased platelet count, decreased white blood cell count, oedema, asthenia, and pneumonitis, whereas ascites, elevated aspartate aminotransferase, and elevated alanine aminotransferase were notably higher in selpercatinib (Table 4).

**TABLE 4:** RORs and *p*-value for pralsetinib and selpercatinib at PT level.

SOC	PT	Pralsetinib		Selpercatinib		*p*-value	ROR ratio (95%CI)
		N	ROR (95%CI)	N	ROR (95%CI)		
Investigations	Blood creatine phosphokinase increased*	12	13.20 (7.48–23.28)	3	8.08 (2.60–25.11)	0.13	2.58 (0.72–9.17)
	Aspartate aminotransferase increased	8	4.13 (2.06–8.27)	16	20.36 (12.43–33.35)	0.005	0.31 (0.13–0.74)
	Alanine aminotransferase increased	6	2.52 (1.13–5.62)	15	15.54 (9.34–25.87)	0.002	0.25 (0.10–0.65)
	Liver function test increased	6	4.73 (2.12–10.55)	14	26.99 (15.93–45.74)	0.004	0.171 (0.066,0.447)
	Platelet count decreased	37	6.59 (4.77–9.127)	11	4.91 (2.71–8.89)	0.02	2.20 (1.11–4.35)
	Blood creatinine increased	19	6.61 (4.21–10.38)	9	7.76 (4.03–14.96)	0.45	1.36 (0.61–3.02)
	Electrocardiogram QT prolonged	-	-	8	11.17 (5.57–22.39)	-	-
	Blood pressure increased	53	6.77 (5.16–8.89)	8	2.52 (1.26–5.06)	<0.001	4.44 (2.09–9.40)
	Blood alkaline phosphatase increased	-	-	5	17.12 (7.11–41.22)	-	-
	Blood bilirubin increased	-	-	5	13.35 (5.54–32.14)	-	-
	Myocardial necrosis marker increased*	21	246.41 (158.78–382.41)	-	-	-	-
	Blood sodium decreased	6	7.46 (3.35–16.62)	-	-	-	-
	hyperphosphatemia	3	25.71 (8.26–79.98)	-	-	-	-
	Troponin increased*	3	8.07 (2.60–25.05)	-	-	-	-
Gastrointestinal disorders	Ascites	9	7.37 (3.83–14.19)	18	37.10 (23.28–59.13)	0.003	0.31 (0.14–0.70)
	Dry mouth	28	8.93 (6.15–12.96)	17	13.42 (8.31–21.67)	0.87	1.05 (0.57–1.94)
	Dysphagia*	18	4.87 (3.06–7.74)	8	5.36 (2.68–10.75)	0.39	1.45 (0.62–3.35)
	Gastroesophageal reflux disease*	-	-	5	3.79 (1.57–9.11)	-	-
	Abdominal distension	10	2.27 (1.22–4.23)	5	2.81 (1.17–6.77)	0.65	1.28 (0.44,3.77)
	Constipation	52	5.08 (3.86–6.68)	-	-	-	-
	Diarrhoea	47	1.48 (1.11–1.98)	-	-	-	-
Blood and lymphatic system disorders	Lymphopenia	3	3.78 (1.22–11.73)	3	9.29 (2.99–28.849)	0.58	0.64 (0.13–3.17)
	Anemia	49	6.13 (4.62–8.12)	7	2.13 (1.01–4.48)	<0.001	4.67 (2.10–10.39)
	White blood cell count decreased	55	9.26 (7.09–12.09)	4	4.51 (1.69–12.03)	<0.001	9.29 (3.35–25.79)
	Neutrophil count decreased	15	1.83 (1.10–3.03)	-	-	-	-
Hepatobiliary disorders	Hepatic function abnormal	35	20.36 (14.58–28.43)	17	24.94 (15.45–40.27)	0.34	1.33 (0.74–2.39)
	Hepatotoxicity	-	-	12	24.71 (13.98–43.65)	-	-
Respiratory, thoracic and mediastinal disorders	Chylothorax	-	-	3	204.11 (65.17–639.27)	-	-
	Pneumonitis	27	19.69 (13.47–28.79)	3	5.40 (1.74–16.77)	0.01	5.90 (1.78–19.53)
	Interstitial lung disease	15	6.71 (4.04–11.15)	-	-	-	-
	pulmonary oedema*	13	7.00 (4.06–12.08)	4	5.31 (1.99–14.18)	0.19	2.09 (0.68–6.45)
	Pleural effusion	20	8.27 (5.32–12.83)	12	12.45 (7.05–21.99)	0.86	1.07 (0.52–2.19)
	Pulmonary embolism*	12	3.75 (2.13–6.62)	5	3.84 (1.60–9.25)	0.42	1.54 (0.54–4.39)
	Pneumothorax*	4	5.23 (1.96–13.96)	3	9.77 (3.15–30.36)	0.83	0.85 (0.19–3.82)
	Cough	28	1.95 (1.34–2.82)	-	-	-	-
	Dysphonia*	7	2.58 (1.23–5.41)	-	-	-	-
General disorders and administration site	Fatigue	88	2.25 (1.82–2.79)	25	1.58 (1.06–2.35)	<0.001	2.39 (1.51–3.77)
	Death	72	1.69 (1.34–2.13)	57	3.38 (2.59–4.41)	0.20	0.79 (0.55–1.14)
	Asthenia	64	3.97 (3.10–5.08)	12	1.82 (1.03–3.22)	<0.001	3.59 (1.92–6.72)
	Pyrexia	53	3.33 (2.53–4.36)	22	3.42 (2.24–5.21)	0.079	1.57 (0.95–2.62)
	oedema	64	8.98 (7.00–11.50)	21	8.97 (5.82–13.81)	0.005	2.02 (1.22–3.35)
Nervous system disorders	Hypogeusia*	11	146.79 (80.51–267.62)	-	-	-	-
	Cognitive disorder*	10	4.30 (2.31–8.00)	-	-	-	-
	Balance disorder*	14	3.78 (2.23–6.39)	-	-	-	-
	Hypoesthesia	15	25.40 (15.28–42.24)	-	-	-	-
	Cerebral infarction*	4	4.68 (1.75–12.48)	-	-	-	-
	Hemiparesis*	-	-	3	12.14 (3.91–37.72)	-	-
Vascular disorders	Hypertension	73	7.51 (5.95–9.48)	18	4.56 (2.86–7.26)	<0.001	2.73 (1.61–4.63)
	Deep vein thrombosis*	-	-	3	3.99 (1.29–12.39)	-	-
Renal and urinary disorders	Renal impairment	33	7.35 (5.21–10.35)	-	-	-	-
	Renal function test abnormal	3	12.40 (3.99–38.51)	-	-	-	-
Metabolism and nutrition disorders	Decreased appetite	22	1.97 (1.30–3.00)	9	2.00 (1.04–3.86)	0.25	1.58 (0.72–3.45)
	Hypocalcemia	4	7.77 (2.91–20.74)	4	12.02 (4.50–32.08)	0.78	0.64 (0.16–2.56)
Musculoskeletal and connective tissue disorders	Rhabdomyolysis*	4	2.73 (1.03–7.29)	-	-	-	-
Cardiac disorders	Myocardial injury*	3	27.75 (8.92–86.36)	-	-	-	-
	pericardial effusion*	-	-	5	12.15 (5.05–29.26)	-	-
Skin and Subcutaneous tissue disorders	Rash	-	-	29	3.44 (2.38–4.98)	-	-

* Indicates signals not documented in the drug label.

Stratified analysis reveals that female treated with pralsetinib experience hypertension, pulmonary embolism, and blurred vision more frequently than men. Conversely, male face a greater risk of rhabdomyolysis. Individuals between 18 to 65 years old tend to develop taste disorders, edema, and pulmonary embolism more often than those older than 65, with an increased susceptibility to hypertension (Table 5).

**TABLE 5:** Stratified analysis of pralsetinib related adverse events.

PT	Gender			Age		
	Male	Female	*p*	18∼65	≥65	*p*
	N (%) = 343	N (%) = 452		N (%) = 262	N (%) = 262	
Hypertension	33 (9.62)	85 (18.81)	<0.001	33 (12.60)	53 (20.23)	0.018
Pneumonia	29 (8.45)	33 (7.30)	0.548	21 (8.02)	27 (10.31)	0.364
Rhabdomyolysis	3 (0.87)	0	0.046	3 (1.15)	0	1.000
Ascites	3 (0.87)	5 (1.11)	0.746	0	0	-
Myocardial necrosis marker increased	6 (1.75)	13 (2.88)	0.303	9 (3.44)	7 (2.67)	0.612
Cognitive disorder	6 (1.75)	4 (0.88)	0.279	0	5 (1.91)	0.072
Taste disorder	15 (2.33)	29 (6.42)	0.212	19 (7.25)	8 (3.05)	0.030
Blood creatinine increased	8 (2.33)	8 (1.77)	0.576	8 (3.05)	8 (3.05)	1.000
Platelet count decreased	10 (2.92)	21 (4.65)	0.212	11 (4.20)	10 (3.82)	0.824
Renal impairment	13 (3.79)	15 (3.32)	0.721	12 (4.58)	9 (3.44)	0.504
Anaemia	25 (7.29)	46 (6.42)	0.157	24 (9.16)	26 (9.92)	0.766
White blood cell count decreased	17 (4.95)	30 (6.64)	0.320	21 (8.02)	17 (6.49)	0.500
Diarrhoea	18 (5.25)	28 (6.19)	0.758	0	0	-
Constipation	23 (6.71)	27 (5.97)	0.674	14 (5.34)	19 (7.25)	0.369
Death	43 (12.54)	0	<0.001	23 (8.78)	0	<0.001
Fatigue	34 (9.91)	53 (11.73)	0.417	25 (9.54)	33 (12.60)	0.265
Pyrexia	27 (7.87)	24 (5.31)	0.144	17 (6.49)	19 (7.25)	0.730
Asthenia	24 (6.99)	36 (7.96)	0.609	19 (7.25)	27 (10.31)	0.217
Pleural effusion	6 (1.75)	10 (2.21)	0.645	7 (2.67)	7 (2.67)	1.000
Oedema	19 (5.54)	29 (6.42)	0.607	25 (9.54)	4 (1.53)	<0.001
Pulmonary embolism	0	10 (2.21)	0.014	6 (2.29)	0	0.040
Interstitial lung disease	0	7 (1.55)	0.053	3 (1.15)	5 (1.91)	0.722
Cerebral infarction	0	3 (0.66)	0.354	0	0	-
Musculoskeletal pain	0	3 (0.66)	0.354	0	0	-
Blood creatine phosphokinase increased	5 (1.46)	6 (1.33)	1.000	6 (2.29)	4 (1.53)	0.523
Vision blurred	0	13 (2.88)	0.002	7 (2.67)	5 (1.91)	0.559

Our study also detected a total of 54 signals of selpercatinib induced AEs across 14 SOCs. Numerous AEs have been documented in clinical trials, such as oedema, fatigue, high blood pressure, dry mouth, skin irritation, low lymphocyte count, raised Alanine Transaminase (ALT) and Aspartate Aminotransferase (AST) levels, aligning with our research findings. In addition, our analysis of data uncovered several AEs that are not currently included on the product label, including pericardial effusion (ROR = 12.15), deep vein thrombosis (ROR = 3.99), pulmonary embolism (ROR = 3.84), dysphagia (ROR = 5.36), gastroesophageal reflux disease (ROR = 3.79), pneumothorax (ROR = 9.77), hemiparesis (ROR = 12.14), and elevated blood creatine phosphokinase levels (ROR = 8.08). Stratified analysis demonstrated that male patients treated with selpercatinib have a significantly higher incidence of fatigue, QT interval prolongation, urinary tract infections, and dysphagia compared to females. Patients aged 18–65 exhibit a significantly higher likelihood of fever and pleural effusion compared to those over 65 years of age, who in turn show a marked increase in hypersensitivity reactions (Table 6).

**TABLE 6:** Stratified analysis of selpercatinib related adverse events.

PT	Gender			Age		
	Male	Female	*p*	18∼65	≥65	*p*
	N (%) = 228	N (%) = 271		N (%) = 128	N (%) = 150	
Fatigue	11 (4.82)	0	<0.001	0	0	-
Rash	10 (4.39)	16 (5.90)	0.447	9 (7.03)	7 (4.67)	0.399
Hypertension	11 (4.82)	9 (3.32)	0.394	6 (4.69)	9 (6.00)	0.629
Death	36 (15.79)	18 (6.64)	0.001	13 (10.16)	23 (15.33)	0.200
Pyrexia	9 (3.95)	10 (3.69)	0.881	7 (5.47)	0	0.012
Ascites	7 (3.07)	3 (1.11)	0.119	0	0	-
Electrocardiogram QT prolonged	6 (2.63)	0	0.007	0	3 (2.00)	0.305
Blood creatinine increased	4 (1.75)	5 (1.85)	0.940			-
Pleural effusion	4 (1.75)	8 (2.95)	0.384	6 (4.69)	0	0.023
Aspartate aminotransferase increased	7 (3.07)	7 (2.58)	0.743	6 (4.69)	5 (3.33)	0.564
Oedema	6 (2.63)	11 (4.06)	0.381	3 (2.34)	3 (2.00)	1.000
Alanine aminotransferase increased	6 (2.63)	6 (2.21)	0.762	5 (3.91)	5 (3.33)	1.000
Dry mouth	6 (2.63)	9 (3.32)	0.653	5 (3.91)	5 (3.33)	0.798
Platelet count decreased	9 (3.95)	14 (5.17)	0.581	8 (6.25)	6 (4.00)	0.393
Dysphagia	4 (1.75)	0	0.029			-
Urinary tract infection	4 (1.75)	0	0.029			-
Hypersensitivity	3 (1.32)	0	0.058	0	6 (4.00)	0.022
Gastrooesophageal reflux disease	3 (1.32)	0	0.058			-
Pulmonary embolism	0	4 (1.48)	0.181			-
Hepatic function abnormal	10 (4.39)	21 (7.75)	0.121	5 (3.91)	9 (6.00)	0.426
Pulmonary oedema	0	3 (1.11)	0.311			
Anaemia	0	0	-	3 (2.34)	0	0.193

### 3.3 Time-to-onset analysis

**FIGURE 2:**
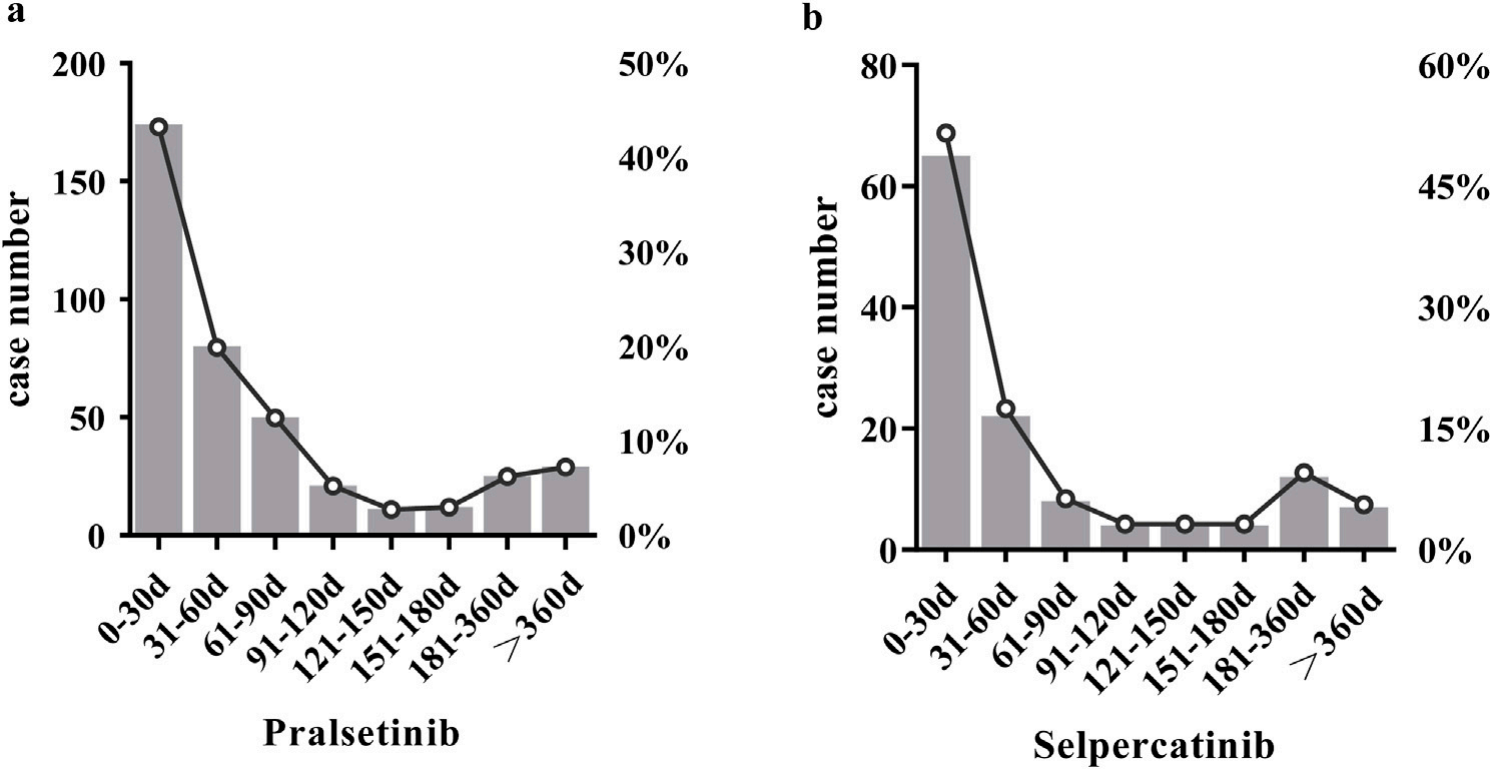
Comparison of onset time of pralsetinib and selpercatinib related adverse events.

We eliminated cases with imprecise, absent, or undisclosed onset times and evaluated 402 pralsetinib-related and 126 selpercatinib-related AE reports with recorded onset times. As illustrated in Figure 2, over 70% of pralsetinib-related AEs manifested within the initial 3 months of treatment commencement, presenting a median onset time of 40 days (IQR14-87.00). For selpercatinib, the median onset time was 29 days (IQR 13–92.25). Comparatively, the median times to AE onset for pralsetinib and selpercatinib were not statistically different (Days: 29 vs 40, *p* = 0.505). The onset pattern was similar for selpercatinib, with most AEs occurring within the first 3 months.

## 4 Discussion

To our knowledge, this is the most comprehensive and organized analysis of AEs related to pralsetinib and selpercatinib after they have entered the market, using information from the FAERS database. Our findings offer a more detailed and exhaustive depiction and categorization of AEs linked to these two drugs. We found a higher incidence of AEs in female patients over 65 attributed to pralsetinib and selpercatinib, mainly due to the prevalent lung cancer in this demographic (Siegel et al., 2024). Moreover, the notable differences in findings could stem from factors including off-label use of pralsetinib and the advanced age of patients. However, it's plausible that differences observed could also stem from single patients reporting multiple outcomes.

Pralsetinib and selpercatinib share four common SOC, which include investigations, gastrointestinal issues, blood and lymphatic system problems, and hepatobiliary disorders (Table 3). The findings are consistent with the safety data reported in the drug label and clinical trials for both medications (Drilon et al., 2023; Hadoux et al., 2023; Ke et al., 2023). Significantly, in the SOC of investigations, the AE with the most frequent occurrence for pralsetinib was bone marrow suppression, which encompassed reduced platelet count, diminished white blood cell count, and anemia. The most frequently reported AEs associated with selpercatinib were increased levels of ALT and AST. The rate of bone marrow suppression with pralsetinib was notably greater than that with selpercatinib, aligning with the findings of the severe outcome. QT interval prolongation as an AE was observed exclusively in patients receiving selpercatinib; no such AEs were reported for the pralsetinib cohort, consistent with drug label and clinical studies (Nguyen and Geirnaert, 2023; Bradford et al., 2021). Hence, before starting patients on selpercatinib, it is important to evaluate the use of QT-prolonging agents such as venlafaxine, amiodarone, sotalol, clarithromycin, and fluconazole, because of the potential for increased risk of drug-induced QT prolongation.

AEs related to gastrointestinal system disorder such as dry mouth, abdominal distension, and ascites have been reported for both pralsetinib and selpercatinib. Chylothorax was exclusively found in individuals who received selpercatinib, while no AEs linked to chylothorax were seen in those who received pralsetinib. In a study, it was discovered that approximately 7% of patients in the selpercatinib group experienced chylous ascites due to selective RET inhibitors, whereas no AEs were observed in the pralsetinib group (Kalchiem-Dekel et al., 2022). According to literature, both pralsetinib and selpercatinib have been reported to cause ascites in patients (Fricke et al., 2023). Chylous ascites, a highly uncommon side effect observed in individuals taking RET-inhibitors, can sometimes be mistaken for disease advancement. The cause of chylous ascites in individuals receiving RET-inhibitors is not well comprehended. Of note, neither constipation nor diarrhea, as indicated on the drug labels, met the requirements for significant RORs of selpercatinib.

Blood and lymphatic system disorders are frequent found during RET-inhibitors treatment, including anemia, lymphopenia, leukopenia and thrombocytopenia, of which anemia is the most common event. Our results indicate that the anemia caused by pralsetinib is significantly higher than that of selpercatinib (ROR = 4.67, *p* < 0.001). In clinical practice, RET-inhibitors are associated with severe safety concerns related to the hepatobiliary system. In our study, the ROR values are statistically significant for hepatic disorders in selpercatinib but not in pralsetinib at PT levels. Elevated alanine aminotransferase and aspartate aminotransferase are more common in the selpercatinib treatment group, with significant statistical differences in results (ROR = 0.25, *p* = 0.002; ROR = 0.31, *p* = 0.005).

Although no significant difference was found in respiratory, thoracic and mediastinal disorders between pralsetinib and selpercatinib, pneumonia is an AE that may occur during the treatment of both pralsetinib and selpercatinib. Our research indicated a higher rate of pneumonia in individuals treated with pralsetinib compared to selpercatinib (ROR = 5.90, *p* = 0.01). Pneumonia is a AE of RET inhibitor; it is also one of the cause of dose modification, interruption, and discontinuation. During the ARROW study, 12.1% of individuals with non-small cell lung cancer experienced treatment-induced pneumonitis, with severe events classified as grade 3 to 4 occurring in 2.1% of cases (Gainor et al., 2021). Accordingly, during the administration of pralsetinib, it is imperative to intensify patient surveillance and ensure prompt intervention upon the manifestation of clinical symptoms. Literature indicates that patients who initially respond to pralsetinib but later experience lung inflammation could potentially see improvements with selpercatinib treatment (d’Arienzo et al., 2023; Cognigni et al., 2024).

Drug-induced rhabdomyolysis is a rare and severe AE that involves muscle damage and multiple-organ failure (Wen et al., 2019). The lack of comprehensive understanding and data on the pathophysiology and progression of rhabdomyolysis hinders the development of early biomarkers and prevention tactics. According to our knowledge, there are no reports of rhabdomyolysis caused by pralsetinib. Multiple cases of tyrosine kinase inhibitors (TKIs)-induced rhabdomyolysis have been reported. One such case was reported by Li et al., where osimertinib was found to be the cause of rhabdomyolysis (Li et al., 2023). Additionally, Obayashi S et al. reported a case of gefitinib-induced rhabdomyolysis (Obayashi et al., 2022). In our study, rhabdomyolysis as an AE was only found induced by pralsetinib. Currently, mechanisms underlying rhabdomyolysis induced by pralsetinib remain unknown. Our research identified an AE of elevated markers of myocardial necrosis, exclusive to patients receiving pralsetinib. Hence, monitoring and follow-up before, during, and after pralsetinib treatment are crucial to mitigate adverse cardiac reactions. Moreover, to minimize the risk of potential AEs, the co-administration of interacting drugs during pralsetinib therapy should not be overlooked.

Pralsetinib is a new type of TKI created to specifically block RET-kinase activity. Although pralsetinib is associated with hypertension and cytopenias, systolic dysfunction has not been recognized as a frequent AE by the FDA. Cardiotoxicity is linked to TKIs, particularly newer ones that block the powerful angiogenic factor vascular endothelial growth factor (VEGF) and disrupt heart function (Chen et al., 2008; Touyz and Herrmann, 2018). Theoretically, pralsetinib’s preference for RET-kinase lowers the chance of heart toxicity commonly linked with nonselective TKIs and VEGF blockage (Chen et al., 2008). Nevertheless, pralsetinib has shown effectiveness in laboratory tests against VEGFR-2, Janus kinase (JAK) inhibitors, and various other substances (Papaila and Jacobson, 2021). The partial specificity towards RET kinase may lower the chance of cardiotoxic effects, but does not completely eliminate it (Chen et al., 2008). Our study uncovered an AE of myocardial injury caused by pralsetinib treatment, which is not included in the drug label. Papaila A et al. reported an AE of myocardial lesions caused by pralsetinib treatment (Papaila and Jacobson, 2021). In addition to strengthening monitoring and evaluating cardiac function, cancer patients treated with pralsetinib usually require the involvement of a multidisciplinary team once related adverse cardiac reactions occur.

AEs on mood and cognition (MCAEs) caused by drugs are frequently identified only during clinical trials or post-marketing, presenting a significant safety issue and difficulty for healthcare providers. Our study found that cognitive impairment was only present in patients treated with pralsetinib. As far as we know, there have been no documented cases of cognitive decline resulting from RET inhibitors. Studies have shown that medications that do not affect the central nervous system much can still cause MCAEs by disrupting the peripheral immune system (Andronis et al., 2020).

Furthermore, selpercatinib exhibited a higher ROR for pericardial effusion, a finding not previously documented in studies. Additionally, pulmonary embolism has been reported as an AE for selpercatinib, despite not being included in the product labels. Our research found that pulmonary embolism was recognized as a novel and important AE linked to selpercatinib, showing signal strengths of ROR = 3.84. Clinical trials have found that pralsetinib can cause AEs to pulmonary embolism, but no related AEs have been found in the population treated with selpercatinib (Hadoux et al., 2023; Gainor et al., 2021). Further observation and research are needed.

Deep vein thrombosis can significantly impact quality of life, yet the drug label fails to mention this AE. Additionally, significant signals of deep vein thrombosis AEs were detected for selpercatinib (ROR = 3.99). The occurrence of thrombosis in patients does not rule out that it is related to the hypercoagulable state of blood in tumor patients, which is worthy of further observation and research in the future.

The relationship between gender, age, and AEs associated with pralsetinib and selpercatinib does not always align with their pharmacokinetic properties; patient-specific variability may play a role. Studies indicate that the transporters ATP-binding cassette sub-family B member 1 and ATP-binding cassette sub-family G member 2 limit the oral bioavailability and cerebral absorption of the RET inhibitors pralsetinib and selpercatinib (Wang et al., 2021a; Şentürk et al., 2020; Wang et al., 2021b). Gender-based variabilities in AEs may be partly attributable to individual patient differences. Clinicians should consider these findings and further validation is warranted in future research.

There was no statistically significant difference in the median time of AEs between pralsetinib and selpercatinib, which were 40 days and 29 days, respectively. The majority of AEs both occurring within the first 3 months following drug initiation. This underscores the crucial need to monitor for AEs during the initial 3 months of drug treatment. Therefore, in future clinical studies, closer monitoring of patients is required to determine the AEs of pralsetinib and selpercatinib.

Our research has several limitations. Initially, the act of willingly submitting information to the database leads to inconsistencies in the quality of data. Noteworthy alterations in how drugs are given, as well as mistakes in numbers, units, and other factors, create difficulties in accurately determining drug dosages and conducting sensitivity analyses. Moreover, our analysis did not consider several unmeasured confounders like drug-drug interactions, polypharmacy, and comorbidities that could affect AE occurrence. Third, due to the unavailability of information on different ethnicities in the FAERS database, this study cannot further analyze the differences in adverse reactions among various ethnic groups. Fourth, the incidence of drug-related AEs cannot be precisely estimated because of insufficient details on patients exposed to the drug and thus without AEs. Additionally, establishing a direct causal link between the drug and the AEs observed is not feasible. Disproportionality analysis assesses signal strength, which can be statistically significant, yet it doesn't quantify risk levels or confirm causality. Therefore, it is essential to conduct larger clinical trials in the future to validate the relationship between the medication and AEs.

## 5 Conclusion

Our study investigated the safety profiles of the RET inhibitors pralsetinib and selpercatinib using the FAERS database. Common AEs linked to RET inhibitors consist of fatigue, hypertension, dry mouth, low red blood cell count, oedema, reduced white blood cell count, and increased ALT and AST levels. Notably, pralsetinib showed significantly higher signal strengths for fatigue, hypertension, pneumonitis, anemia, decreased platelet count, asthenia and decreased white blood cell count at the PT level than selpercatinib. Conversely, selpercatinib had significantly higher signal strengths for ascites, increased ALT, and elevated AST compared to pralsetinib. Additionally, our findings suggest that pneumothorax and increased blood creatine phosphokinase, not listed in the drug labels, should be considered when using these medications. At the PT level, we identified significant signals, such as rhabdomyolysis, myocardial injury, and cognitive disorder associated with pralsetinib, and pulmonary embolism, deep vein thrombosis and pericardial effusion associated with selpercatinib. These AEs are not listed in the drug package inserts. In the course of pralsetinib treatment, female exhibit a higher incidence of hypertension, pulmonary embolism, and blurred vision than male, who in turn show a greater susceptibility to rhabdomyolysis. Adults between 18 to 65 years old have a greater tendency to experience taste disorders, edema, and pulmonary embolism compared to those over 65, with a notably higher risk of developing hypertension. During selpercatinib therapy, male patients are more prone to significant increases in fatigue, QT prolongation on electrocardiograms, urinary tract infections, and dysphagia. Individuals aged 18 to 65 tend to have higher incidences of fever and pleural effusion compared to those over 65, who are more susceptible to hypersensitivity reactions. In conclusion, our study offers further insights into the safety of pralsetinib and selpercatinib. Nonetheless, additional research is required to clarify the underlying mechanisms of unexpected AEs and to confirm their causative links with these medications. Moreover, continued investigation is imperative to explore how age and gender influence specific AEs to RET inhibitors.

## Data Availability

The datasets presented in this study can be found in online repositories. The names of the repository/repositories and accession number(s) can be found in the article/Supplementary Material.
