# Supplementary material for: Adverse event profile differences between pralsetinib and selpercatinib: a real-world study based on the FDA adverse events reporting system
Source: Front Pharmacol. 2024 Sep 20;15:1424980. doi: 10.3389/fphar.2024.1424980 (PMC11449734; doi:10.3389/fphar.2024.1424980)
Supplement: Supplementary file 1 [file Table1.docx]

Supplementary Table 1 Signal strength of reports of pralsetinib at the SOC level in the FAERS database

| System organ class (SOC) | Pralsetinib cases reporting SOC | ROR (95%CI) |
| --- | --- | --- |
| **General disorders and administration site conditions#** | 603 | 1.14( 1.04- 1.25)* |
| **Investigations#** | 447 | 2.71( 2.45- 2.99)* |
| **Gastrointestinal disorders#** | 359 | 1.58 ( 1.42- 1.77) )* |
| **Nervous system disorders#** | 248 | 1.14( 1.003- 1.30)* |
| **Respiratory, thoracic and mediastinal disorders#** | 244 | 1.87( 1.64 - 2.13) )* |
| Infections and infestations# | 170 | 0.99( 0.85- 1.16) |
| Musculoskeletal and connective tissue disorders | 129 | 0.80 ( 0.67- 0.96) |
| **Blood and lymphatic system disorders** | 113 | 2.22( 1.84- 2.67) * |
| **Vascular disorders** | 109 | 1.97( 1.62- 2.38) * |
| Injury, poisoning and procedural complications | 108 | 0.26( 0.22- 0.32) |
| **Hepatobiliary disorders** | 77 | 3.23( 2.58- 4.05) * |
| Skin and subcutaneous tissue disorders | 75 | 0.48 ( 0.38- 0.60) |
| **Renal and urinary disorders** | 73 | 1.29 ( 1.02 - 1.62) * |
| Metabolism and nutrition disorders | 67 | 1.17( 0.92- 1.49) |
| Eye disorders# | 57 | 0.98( 0.75- 1.27) |
| Neoplasms benign, malignant and unspecified (incl cysts and polyps) | 54 | 0.36( 0.27- 0.47 ) |
| Psychiatric disorders | 48 | 0.27( 0.20- 0.36) |
| Cardiac disorders | 33 | 0.55 ( 0.39 - 0.78) |
| Surgical and medical procedures | 12 | 0.27 ( 0.15 - 0.48) |
| Reproductive system and breast disorders# | 12 | 0.66 ( 0.38- 1.17) |
| Immune system disorders | 9 | 0.27 ( 0.14 - 0.51) |
| Endocrine disorders | 7 | 0.88 ( 0.42- 1.85) |
| Ear and labyrinth disorders | 6 | 0.49( 0.22- 1.08) |
| Social circumstances | 2 | 0.13 ( 0.03- 0.54) |
| Product issues | 1 | 0.12 ( 0.02- 0.87) |
