# Supplementary figures and images for: Adverse event profile differences between pralsetinib and selpercatinib: a real-world study based on the FDA adverse events reporting system
Source: Front Pharmacol. 2024 Sep 20;15:1424980. doi: 10.3389/fphar.2024.1424980 (PMC11449734; doi:10.3389/fphar.2024.1424980)

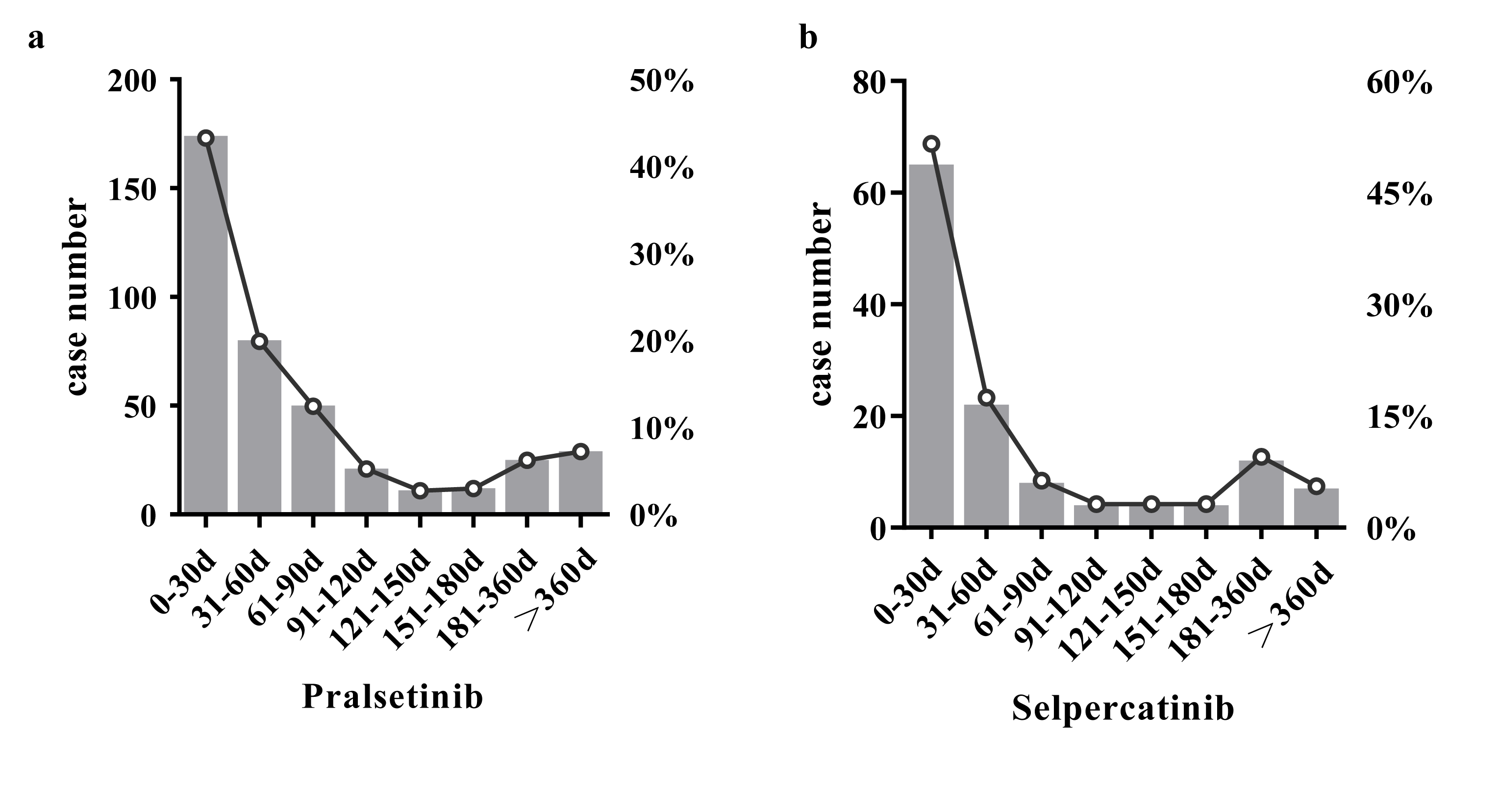

Supplement: Supplementary file 2 [file Image2.TIF]

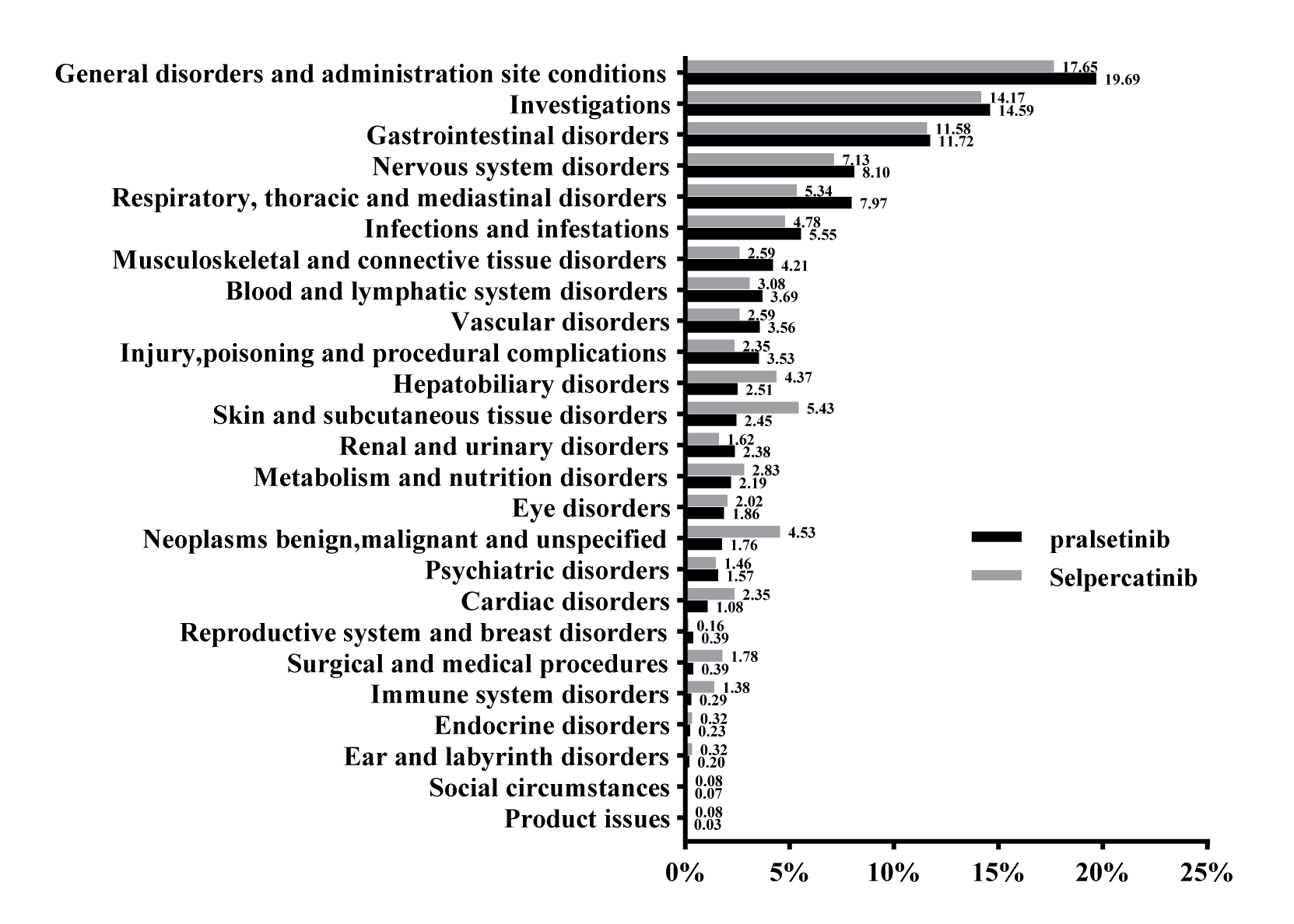

Supplement: Supplementary file 3 [file Image1.TIF]
