# Supplementary material for: Adverse event profile differences between pralsetinib and selpercatinib: a real-world study based on the FDA adverse events reporting system
Source: Front Pharmacol. 2024 Sep 20;15:1424980. doi: 10.3389/fphar.2024.1424980 (PMC11449734; doi:10.3389/fphar.2024.1424980)
Supplement: Supplementary file 4 [file Table2.docx]

Supplementary Table 2 Signal strength of reports of selpercatinib at the SOC level in the FAERS database

| System organ class (SOC) | Selpercatinib cases reporting SOC | ROR (95%CI) |
| --- | --- | --- |
| General disorders and administration site conditions | 218 | 0.99 ( 0.86- 1.15 ) |
| **Investigations** | 175 | 2.62 ( 2.23- 3.07 )* |
| **Gastrointestinal disorders** | 143 | 1.56 ( 1.31 - 1.85 )* |
| Nervous system disorders | 88 | 0.99 ( 0.80 - 1.23 ) |
| Skin and subcutaneous tissue disorders | 67 | 1.04( 0.81 - 1.33 ) |
| Respiratory, thoracic and mediastinal disorders | 66 | 1.21 ( 0.95 - 1.55 ) |
| Infections and infestations | 59 | 0.85 ( 0.65 - 1.10 ) |
| Neoplasms benign, malignant and unspecified (incl cysts and polyps) | 56 | 0.98 ( 0.75- 1.28) |
| **Hepatobiliary disorders** | 54 | 5.68 ( 4.33 - 7.47)* |
| **Blood and lymphatic system disorders** | 38 | 1.84 ( 1.33 - 2.54 )* |
| **Metabolism and nutrition disorders** | 35 | 1.52 ( 1.09- 2.1)* |
| Vascular disorders | 32 | 1.41( 0.99- 2.01) |
| Musculoskeletal and connective tissue disorders | 32 | 0.49( 0.34- 0.69) |
| Cardiac disorders | 29 | 1.21 ( 0.84 - 1.75 ) |
| Injury, poisoning and procedural complications | 29 | 0.17 ( 0.12 - 0.25 ) |
| Eye disorders | 25 | 1.06 ( 0.72- 1.58) |
| Surgical and medical procedures | 22 | 1.26 ( 0.82 - 1.92) |
| Renal and urinary disorders | 20 | 0.86 ( 0.55 - 1.34 ) |
| Psychiatric disorders | 18 | 0.25 ( 0.16- 0.40) |
| Immune system disorders | 17 | 1.24 ( 0.77 - 2.00 ) |
| Endocrine disorders | 4 | 1.25 ( 0.47 - 3.35 ) |
| Ear and labyrinth disorders | 4 | 0.80 ( 0.30 - 2.14) |
| Social circumstances | 2 | 0.49 ( 0.12 - 1.97 ) |
| Reproductive system and breast disorders | 2 | 0.27( 0.07 - 1.07) |
| Product issues | 1 | 0.17 ( 0.023 - 1.19 ) |
